# Synthesis of rare-earth metal compounds through enhanced reactivity of alkali halides at high pressures

**DOI:** 10.1038/s42004-022-00736-x

**Published:** 2022-10-08

**Authors:** Yuqing Yin, Fariia I. Akbar, Elena Bykova, Alena Aslandukova, Dominique Laniel, Andrey Aslandukov, Maxim Bykov, Michael Hanfland, Gaston Garbarino, Zhitai Jia, Leonid Dubrovinsky, Natalia Dubrovinskaia

**Affiliations:** 1 Material Physics and Technology at Extreme Conditions, Laboratory of Crystallography, University of Bayreuth, 95440 Bayreuth, Germany; 2 State Key Laboratory of Crystal Materials, Shandong University, 250100 Jinan, China; 3 Bayerisches Geoinstitut, University of Bayreuth, 95440 Bayreuth, Germany; 4 Earth and Planets Laboratory, Carnegie Institution for Science, Washington, DC 20015 USA; 5 Centre for Science at Extreme Conditions and School of Physics and Astronomy, University of Edinburgh, EH9 3FD Edinburgh, UK; 6 Institute of Inorganic Chemistry, University of Cologne, 50939 Cologne, Germany; 7 European Synchrotron Radiation Facility, F-38043 Grenoble, France; 8 Department of Physics, Chemistry and Biology (IFM), Linköping University, SE-581 83 Linköping, Sweden

**Keywords:** Chemical synthesis, Chemical physics, Inorganic chemistry, Solid-state chemistry

## Abstract

Chemical stability of the alkali halides NaCl and KCl has allowed for their use as inert media in high-pressure high-temperature experiments. Here we demonstrate the unexpected reactivity of the halides with metals (Y, Dy, and Re) and iron oxide (FeO) in a laser-heated diamond anvil cell, thus providing a synthetic route for halogen-containing binary and ternary compounds. So far unknown chlorides, Y_2_Cl and DyCl, and chloride carbides, Y_2_ClC and Dy_2_ClC, were synthesized at ~40 GPa and 2000 K and their structures were solved and refined using in situ single-crystal synchrotron X-ray diffraction. Also, FeCl_2_ with the HP-PdF_2_-type structure, previously reported at 108 GPa, was synthesized at ~160 GPa and 2100 K. The results of our ab initio calculations fully support experimental findings and reveal the electronic structure and chemical bonding in these compounds.

## Abstract (web summary)

The chemical stability of alkali halides has caused them to be exploited as inert media in high-pressure, high-temperature experiments. Here, NaCl and KCl are unexpectedly found to react with yttrium, dysprosium and iron oxide in a laser-heated diamond anvil cell, producing Y_2_Cl, DyCl, Y_2_ClC and Dy_2_ClC at ~40 GPa and 2000 K and FeCl_2_ at ~160 GPa and 2100 K.

## Introduction

Alkali halides, particularly sodium and potassium chlorides, are chemically very stable and are usually not considered as precursors for the synthesis of new compounds in high-pressure (HP) studies. Indeed, NaCl and KCl were thought to be chemically inert over wide pressure (up to 200 GPa) and temperature (up to 3000 K) ranges^1^. Therefore, they have often been used as pressure calibrants^2^, pressure transmitting media^3^, and electrical and thermal insulators in HP experiments^3–5^. Recent experimental and theoretical studies^6–8^ suggest, however, that the behavior of the Na-Cl and K-Cl systems at HP is complex, and several compounds with an unusual stoichiometry (like NaCl_3_, Na_3_Cl, Na_2_Cl, and KCl_3_) have been reported. Still, NaCl and KCl are considered to be chemically stable under HP, as in the absence of ionization-promoting species^9,10^, reactions are found in the presence of extra chlorine or sodium/potassium in a diamond anvil cell (DAC)^7,8^.

Being formed by highly electropositive and electronegative elements, NaCl and KCl, having a stable electron configuration, are not expected to react with transition or rare-earth metals. In the present work we have shown that it is not the case under pressure, as our experiments, originally designed to study the HP behavior of metals (Y, Dy, Re, and Ag) in an “inert” pressure medium (NaCl) in a laser-heated diamond anvil cell (LHDAC), resulted in the synthesis of previously unknown chlorides, Y_2_Cl and DyCl, and chloride carbides, Y_2_ClC and Dy_2_ClC, at about 40 GPa and 2000 K. An iron chloride, FeCl_2_, with the HP-PdF_2_-type structure, was found to be a product of a chemical reaction between FeO and KCl in a LHDAC at about 160 GPa and 2100 K.

Here we report the crystal structures of the chloride phases, Y_2_Cl and DyCl, and chloride carbides Y_2_ClC and Dy_2_ClC, as well as the structure of iron chloride, FeCl_2_. The structures were solved and refined using in situ high-pressure synchrotron single-crystal X-ray diffraction in a DAC. Our ab initio calculations are in good agreement with the experimental results.

## Results and discussion

### Reactivity of alkali halides and heavy metals at high pressures

To conduct the experiments, pieces of metals (Y, Dy, Re, or Ag) or FeO were loaded into a DAC between two layers of dried sodium or potassium chlorides. All experiments were performed in BX90-type DACs equipped with Boehler-Almax type diamond anvils with culets of 250 or 120 μm^11^. NaCl or KCl served as pressure-transmitting media and turned out to also act as reactants. To facilitate a chemical reaction, the samples were laser-heated using YAG lasers with the metals serving as the absorbers.

Supplementary Table 1 provides information on all experiments carried out in this work. Silver was not found to react with NaCl after heating up to ~1950 K at ~44 GPa (Supplementary Fig. 1). Experiments with other metals, for example upon heating Dy in NaCl at ~40 GPa (Supplementary Fig. 2), optical observations provided early signs of chemical reactions. Raman spectroscopy was also helpful in some cases in giving a clear indication of a sample change after heating, like in the experiment with Re laser-heated in NaCl at ~38 GPa (Supplementary Fig. 3). However quite often Raman spectra did not yield any information on the phonon modes of the newly formed solids because of strong luminescence signals coming from the laser-heated spots.

**Fig. 1:**
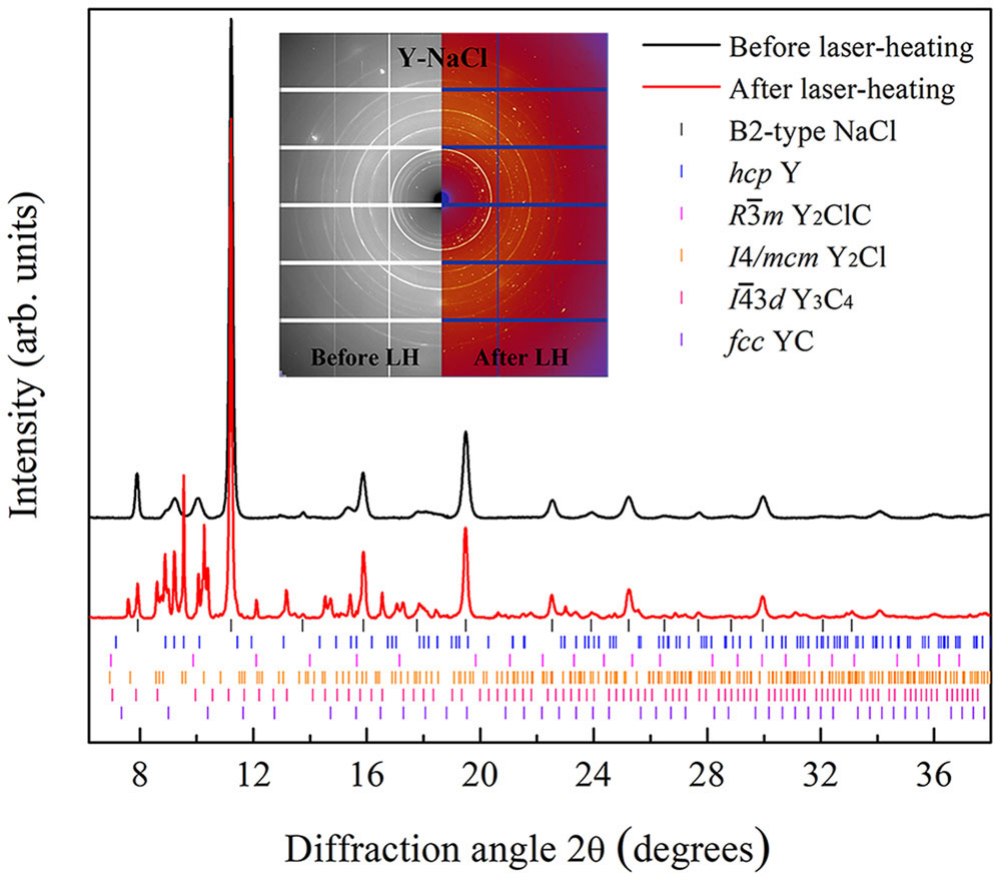
Chemical reactions between yttrium and NaCl detected from X-ray diffraction. Powder X-ray diffraction patterns of the Y-NaCl sample before (black) and after (red) laser-heating at ~2000 K and 41 GPa. Insert shows 2D XRD patterns of the same sample before and after laser heating. The previously known (*fcc*-YC) and unknown (Y_2_Cl, Y_2_ClC, and Y_3_C_4_) compounds were identified using single-crystal XRD data; the ticks here are added according to their calculated powder XRD patterns.

The most reliable and accurate data on chemical reactions were obtained from XRD studies, when a chemical alteration of the sample is manifested in changes in the diffraction pattern and the products of the reactions, their chemical composition and structures, can be characterized using single-crystal X-ray diffraction data. Figure 1 shows powder diffraction patterns of the Y-NaCl sample before and after laser-heating up to ~2000 K at ~41 GPa. The pattern obtained before laser-heating displays only the presence of *hcp*-Y and B2-NaCl in the sample. After heating, additionally to the *hcp*-Y in B2-NaCl reflections, extra diffraction lines were observed (Fig. 1). The 2D XRD patterns in the insert in Fig. 1 show the appearance of numerous diffraction spots after laser heating, characteristic of single crystals. We used our approach to the high-pressure XRD data analysis^12–16^ and the DAFi program^17^, which we specially developed to process single-crystal XRD data from microcrystals. Processing these data revealed the formation of the previously known *fcc*-YC compound^18^, a new HP phase of Y_3_C_4_ (to be published elsewhere), and new yttrium chloride and yttrium chloride carbide (Y_2_Cl and Y_2_ClC), which are discussed in detail in this work. Similarly, chemical reactions were detected in the Dy-NaCl, FeO-KCl, and Re-NaCl systems (Supplementary Table 1 and Supplementary Figs. 1–3), and the new phases, DyCl, Dy_2_ClC, and FeCl_2_, were identified. Unfortunately, despite all our efforts, the new phases in the Re-NaCl system could not be recognized, although we observed the signs of chemical reactions both in Raman spectra and the XRD data (Supplementary Fig. 3). The crystal structures, solved and refined at HP for all phases detected in the Y-NaCl, Dy-NaCl, and FeO-KCl systems after laser heating, are described in detail below.

### Structures of chlorides Y_2_Cl, DyCl, and FeCl_2_

**Fig. 2:**
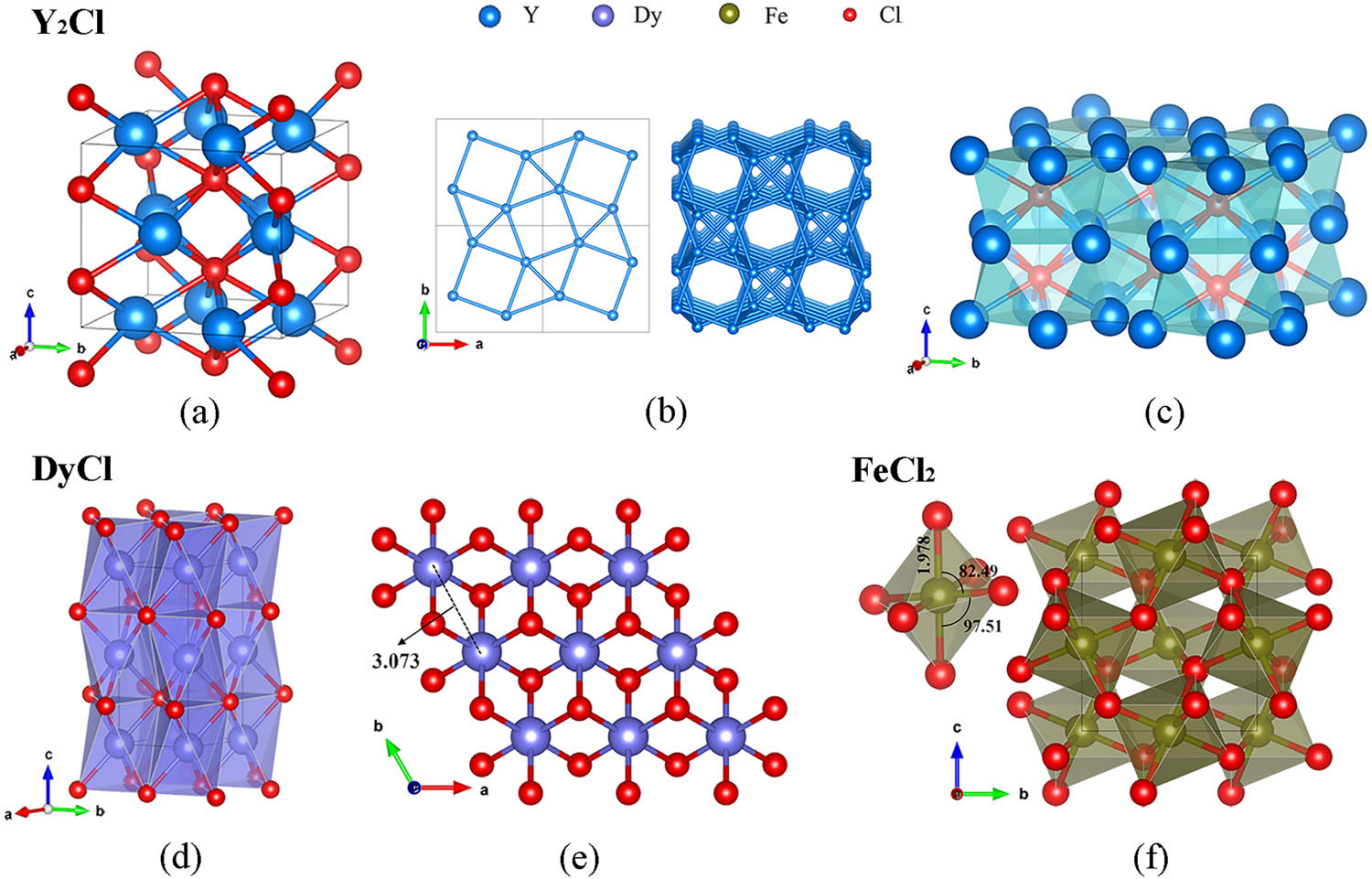
Crystal structures of novel chlorides of Y, Dy, and Fe. **a** Stick-and-ball model of Y_2_Cl structure at 41(1) GPa; **b** view of the Y metal framework in Y_2_Cl along the *c*-direction; **c** square antiprisms geometry around the Cl center in Y_2_Cl. **d** Polyhedral model of the crystal structure of DyCl at 40(1) GPa built of YCl_6_ octahedra; **e** view of the structure along the *c*-direction: Cl atoms form hexagonal close packing (*hcp*) and Y atoms occupy the space in between. **f** Polyhedral model of FeCl_2_ at 160(1) GPa. Y, Dy, Fe, and Cl atoms are shown in blue, purple, tan, and red colors, respectively.

The yttrium-chlorine compound, Y_2_Cl, synthesized at ~41 GPa and ~2000 K (Supplementary Table 1) crystallizes in a structure with the tetragonal space group *I*4*/mcm* (#140). Y atoms occupy the 8 *h* Wyckoff site with the atomic coordinates (0.659(7) 0.159(7) 1/2) while the Cl atoms occupy the 4*a* (0 0 1/4) site (Fig. 2a, see also the CIF deposited at CSD 2184741 and Supplementary Data 1). The lattice parameters are *a* = 6.128(3) Å and *c* = 5.405(7) Å at 41(1) GPa. The full experimental crystallographic data, including the crystal structure, data collection and refinement details of Y_2_Cl at 41(1) GPa are provided in Supplementary Table 2.

Yttrium atoms form 3^2^.4.3.4 nets in the *ab* plane (Fig. 2b). The nets are stacked along the *c* direction with a ½ period and rotated by 90° from one another (Fig. 2b). The chlorine atoms are located in the centers of square antiprisms formed between the layers of Y atoms (Fig. 2c). The Y-Cl distances within the antiprisms (~2.67 Å) are compatible with those known for other yttrium chlorides at ambient conditions (YCl^19,20^, Y_2_Cl_3_^21^, YCl_3_^22^–2.70–2.75 Å). The striking difference is in the Cl-Cl and Y-Y distances (~2.70 Å and ~2.75 Å, correspondingly) of Y_2_Cl compared to the known yttrium chlorides with predominantly ionic bonding between Y and Cl and metal-metal bonding within metal frameworks (for example, 3.30–3.72 Å for Cl-Cl and 3.27–3.96 Å for Y-Y in YCl and Y_2_Cl_3_)^21,23^. This indicates that the “metallic radius”^24^ in Y_2_Cl is different (smaller) from those in other yttrium chlorides. This might contribute to the reduction in enthalpy, making Y_2_Cl a favored phase at HP. In fact, the Y_2_Cl compound was predicted to be metallic at 20 GPa^25^, and in metallic *hcp* yttrium at 40 GPa^26^ the unit cell volume per atom is ~19.9 Å^3^ that allows one to calculate the shortest Y-Y contact to be ~2.7 Å, which matches our measurements. Our ab initio calculations (Methods section) well reproduced the experimental crystal structure of Y_2_Cl at 40 GPa (Supplementary Table 2) and confirmed its dynamical stability (Supplementary Fig. 4).

The structure of dysprosium chloride, DyCl, has a hexagonal unit cell with the lattice parameters *a* = 3.079(19) Å and *c* = 7.621(5) Å at 40(1) GPa (Fig. 2d, e, see also the CIF deposited at CSD 2184740 and Supplementary Data 2), and the space group symmetry *P*6_3_*/mmc* (#194) with the Dy atoms occupying the 2*a* Wyckoff site (0 0 1/2), and Cl atoms occupying the 2*c* site (1/3 2/3 3/4) (Table S3). In this NiAs (B8) type structure, the Cl atoms form a hexagonal close packing (*hcp*), in which Dy atoms occupy the centers of the edge-sharing octahedra. Although there are other rare-earth (RE) chlorides known at atmospheric pressure (RE = Sc, Y, Gd, and La)^23,27,28^, they possess ZrCl-type structure, which is different from that of the HP Dy and Y chlorides.

The calculated structural parameters are in good agreement with the experimental results (Supplementary Table 3). DyCl is dynamically stable at our experimental pressure (~40 GPa) and its metallic nature at 40 GPa is confirmed with the Dy-*d* electrons dispersed at the Fermi level due to Dy-Dy metallic bonding (Supplementary Fig. 5).

The HP phase of FeCl_2_ crystalized at 160(1) GPa in the HP-PdF_2_ type structure with the *P*$a\bar{3}$ (#205) space group and the lattice parameter *a* = 4.829(11) Å (Fig. 2f). Fe atoms occupy the 4*b* Wyckoff site, while Cl atoms occupy the 8*c* site, and form vertex-sharing Cl_6_ octahedra with Fe atoms in the centers. The Fe-Fe, Fe-Cl, and Cl-Cl distances at 160(1) GPa are 3.41 Å, 1.98 Å, and 2.61 Å, respectively.

Our theoretical calculations reproduced the experimental crystal structure of FeCl_2_ at 150 GPa (Supplementary Table 4) and confirmed its dynamical stability down to a pressure of 90 GPa (Supplementary Fig. 6). The measured unit cell volume and the pressure-volume points from theoretical calculations are shown in Supplementary Fig. 7. The calculated bulk modulus of *K*_0_ = 96.9(14) GPa (*K*′ = 4.45(2); *V*_0_ = 185.5(4) Å^3^) was determined by fitting the third-order Birch-Murnaghan equation of state to the calculated P-V data (Supplementary Fig. 7). The calculated band structure along specific high-symmetry directions suggests that the cubic FeCl_2_ phase is a typical example of normal semimetals^29^, in which electron and hole pockets coexist on the Fermi surface (Supplementary Fig. 8).

It is worth mentioning that at ambient conditions FeCl_2_ (phase I) possesses the layered CdCl_2_-type structure (*R*$\bar{3}$*m*, #166), in which the chlorine atoms form a cubic close packing (*ccp*). Fe atoms, which fill ½ of its octahedral voids, are “sandwiched” between the two sheets of chlorine atoms^30^, producing Cl-Fe-Cl layers separated from each other. At low pressures (~0.6 GPa) phase I undergoes a structural transition to phase II with the hexagonal CdI_2_-like structure (*P*$\bar{3}$*m1*, #164), which is similar to that of phase I, but in phase II the chlorine atoms form a hexagonal close packing (*hcp*). This structure persists to 65 GPa^31^. At 108 GPa and 2000 K Yuan et al.^9^ synthesized a FeCl_2_ phase with the same HP-PdF_2_ type structure, which we observed in this work at 160 GPa, but the synthesis was realized through hydrous systems to force the ionization of NaCl.

### Structures of novel chloride carbides Y_2_ClC and Dy_2_ClC

As shown in previous work^32^, carbon from diamond anvils, being mobilized upon laser heating, can participate in chemical reactions. In this work, this phenomenon has led to the synthesis of previously unknown ternary compounds, Y_2_ClC and Dy_2_ClC, which are isostructural (space group *R*$\bar{3}$*m*, #166) and with similar lattice parameters. Y and Dy atoms occupy the Wyckoff site 6*c*, Cl atoms occupy the 3*a* site, and C atoms occupy the 3*b* site. The full experimental crystallographic data, including the crystal structure, data collection, and refinement details for these phases are provided in Supplementary Tables 5 and 6 (see also the CIF deposited at CSD 2184739 and 2184742) (Supplementary Data 3 for Y_2_ClC and Supplementary Data 4 for Dy_2_ClC).

**Fig. 3:**
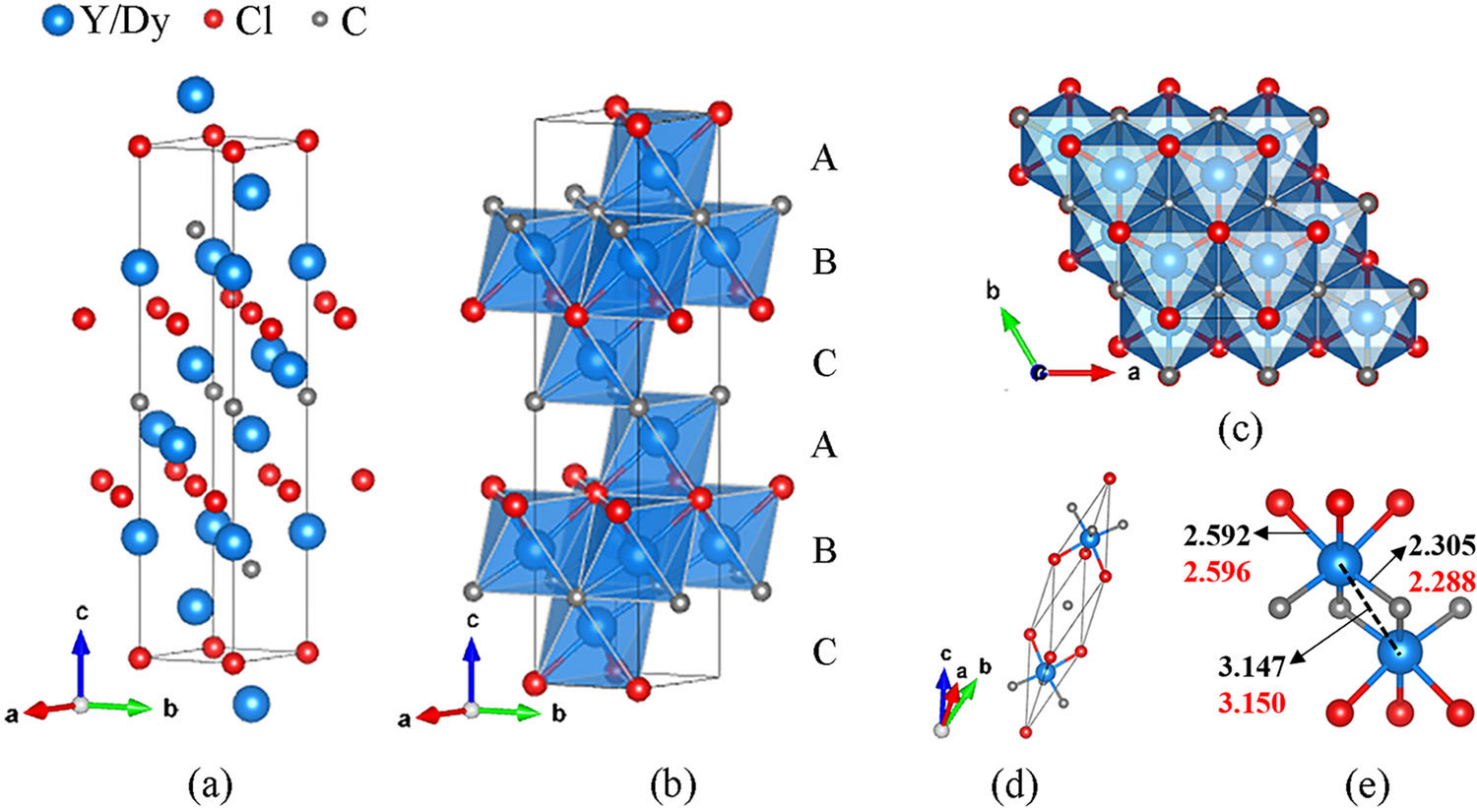
Crystal structure of Y_2_ClC and Dy_2_ClC synthesized at ~40 GPa. **a** Ball model in a hexagonal setting. **b** Polyhedral model built of (YC_3_Cl_3_) octahedra; A, B, C letters highlight the *ccp* formed by Y/Dy atoms. **c** View of the structure along the *c*-direction; C and Cl atoms together forming the *ccp*. **d** Crystal structure in the rhombohedral setting. **e** Interatomic distances (in Å) in Y_2_ClC (black numbers) and Dy_2_ClC (red numbers). Y/Dy, Cl, and C atoms are shown in blue, red, and gray colors.

In the structure of the novel chloride carbides, the rare-earth atoms (Y and Dy) form a distorted cubic close packing (*ccp*) (Fig. 3). If one considers Cl and C as equal-size spheres, the alternating close-packed layers of C and Cl also form a distorted *ccp*. Thus, the structure can be described as one derived from NaCl (B1) type with carbon and chlorine atoms forming the *ccp*, whose octahedral voids are occupied by the rare-earth atoms. Each rare-earth atom is connected to three Cl and three C atoms (Fig. 3d). As shown in Fig. 3e, the metal-C contacts (Y-C ~2.30 Å, and Dy-C ~2.29 Å) are significantly shorter than the metal-Cl ones (Y-Cl ~2.59 Å, and Dy-Cl ~2.60 Å), as expected due to the smaller ionic radius for C atoms. But the Y-Y and Dy-Dy contacts between layers AB, CA, and BC (Fig. 3b) are relatively short (~3.15 Å), and this distance is close to that of Dy-Dy in DyCl with a metallic bonding character, as discussed above.

To gain a deeper insight into the properties of Y_2_ClC and Dy_2_ClC, we performed calculations based on density functional theory (DFT). The relaxed structural parameters (Supplementary Tables 5 and 6) closely reproduce the corresponding experimental values at 40 GPa. Harmonic phonon dispersion calculations using the Phonopy software^33^ show no imaginary frequencies, demonstrating the dynamical stability of *R*$\bar{3}$*m* Y_2_ClC at both 40 GPa and 1 bar (Supplementary Fig. 9). Ohmer et al.^34^ predicted the stability of a Y_2_ClC solid with a *P*6_3_*/mmc* space group (#194), which is considered to be a MAX-type (M_n+1_AX_n_)^35,36^ compound. By comparing the enthalpy values of the two phases (Supplementary Table 7), we suggest that Y_2_ClC will have a phase transition from *R*$\bar{3}$*m* to *P*6_3_*/mmc* when the pressure is reduced to 10 GPa (Supplementary Fig. 9c). No competing phase was found for *R*$\bar{3}$*m* Dy_2_ClC, and its dynamical stability at 40 GPa is demonstrated in Supplementary Fig. 10.

**Fig. 4:**
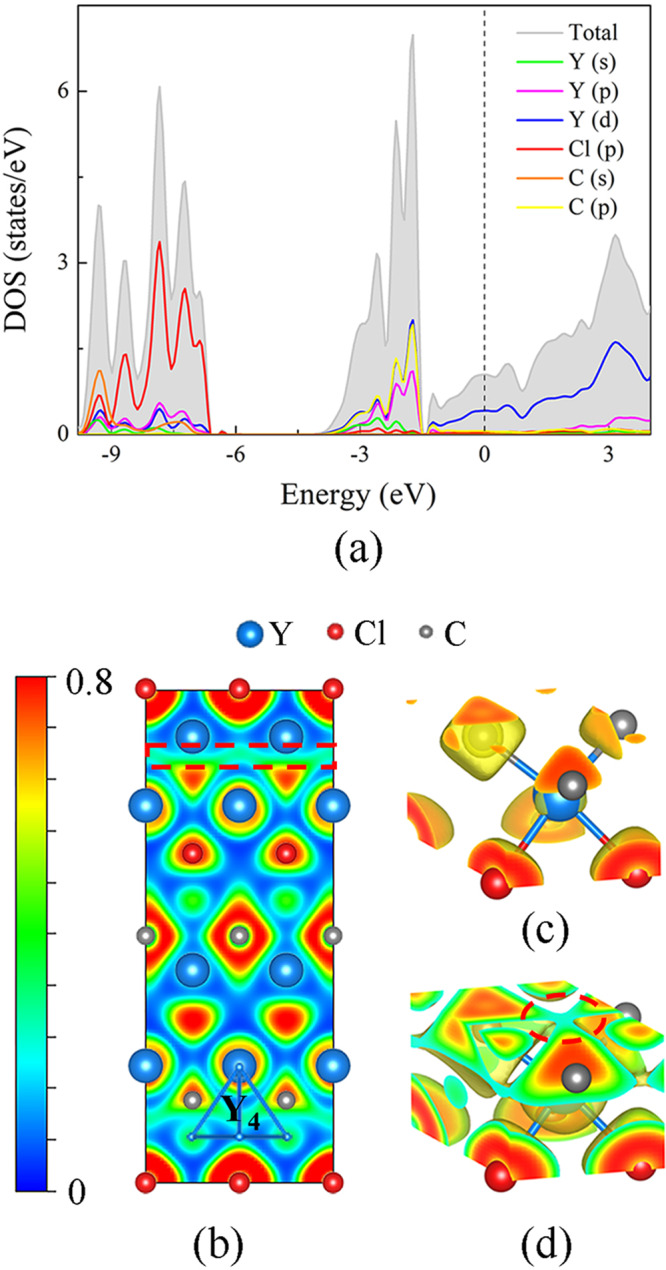
Results of theoretical calculations. **a** The total and projected densities of states (TDOS and PDOS) curves of Y_2_ClC at 40 GPa; the Fermi energy level was set to 0 eV. **b** The 2D electron localization function (ELF) map of Y_2_ClC in the (1 0 0) plane; **c**, **d** ELF with the isosurfaces value set as **c** 0.7 and **d** 0.25. Y, Cl, and C atoms are shown in blue, red, and gray colors. One Y_4_ tetrahedron is highlighted in 4b, and the weak ELF value in its center is highlighted with the red dashed circle in **d**. These weak ELF values in the centers of the Y_4_ tetrahedra form bridges connecting the C atoms (highlighted with the red dashed square in **b**).

Considering that Y_2_ClC and Dy_2_ClC are isostructural, the computed total and projected electron densities of states (TDOS and PDOS) are illustrated in Fig. 4a taking Y_2_ClC as an example. At 40 GPa, Y_2_ClC is a metal, as it shows a non-zero density of states at the Fermi level, and the main contribution at the Fermi level comes from the yttrium *d*-states. Interestingly, the calculated electron localization function (ELF) of Y_2_ClC at 40 GPa not only gives evidence of ionic bonding between the Y-Cl and Y-C atoms but features weak ELF values in the centers of the Y_4_ tetrahedra (Fig. 4b–d), forming bridges connecting the C atoms (see the highlighted red dashed lines in Figs. 4b, d). One can speculate from the ELF values that these ELF bridges are caused by the hybridization of the Y-*d* orbitals (for ELF < 0.5, the metal bonding is undoubtedly more pronounced^37^). To confirm this conclusion, additional DFT calculations were performed with the C atoms removed, resulting in a stable Y_2_Cl electride with a more localized ELF attractor at the center of the Y_4_ tetrahedra and anionic electrons localized at the centers of Y_6_ octahedra (Supplementary Fig. 11). The introduction of C atoms causes a charge loss in the Y_4_ tetrahedra and a charge gain in the Y_6_ octahedra (Supplementary Fig. 11g) resulting in the weak ELF bridges in Fig. 4b. The PDOS of Y-*d* orbitals and the partial charge density map further confirmed the Y-*d* orbital overlapping in the Y_4_ tetrahedra of Y_2_ClC (Supplementary Fig. 11f). Detailed information and further discussion of the electronic properties of Y_2_ClC and Dy_2_ClC can be found in Supplementary Discussion, Supplementary Figs. 11–13 and Supplementary Table 9.

To summarize, the chemical reactions between NaCl and Y, Dy, and Re at ~40 GPa and KCl and FeO at ~160 GPa, observed in the present work under HP, were unexpected. They led to the synthesis of hitherto unknown chlorides, Y_2_Cl and DyCl, and chloride carbides, Y_2_ClC and Dy_2_ClC. Although these results limit the application of alkali halides as thermal insulators and pressure media in LHDACs, as reactants, they provide a surprisingly simple route for the preparation of halogen-containing compounds.

## Methods

### Sample preparation

One stack of halide (NaCl or KCl) thin (3–5 µm) plate was first loaded on one of the diamond anvils, with a culet diameter of 250 µm (for DAC 1–4) or 120 µm (for DAC 5; Supplementary Table 1). A piece of pure flake of metal (Y/Dy/Re/Ag) or FeO of typically ~5 × 5 × 5 µm^3^ in size was positioned on the halide layer. Then we placed another stack of halide (NaCl or KCl) thin (3–5 µm) plate on the other diamond anvil so that the samples were loaded as sandwiches. Rhenium was used as the gasket material. NaCl and KCl powders were dried on a heating table at 220 °C for 48 h before loading to avoid any presence of water. The in situ pressure was measured using the first-order Raman mode of the stressed diamond anvils^38^. Double-sided sample laser-heating was performed at our home laboratory at the Bayerisches Geoinstitut^39^. Detailed information of pressure and the heating temperature can be found in Supplementary Table 1.

### X-ray diffraction

Synchrotron X-ray diffraction measurements of the compressed samples were performed at ID15 (*λ* = 0.41015 Å, beam size ~5.0 × 5.0 μm^2^) and ID27 (*λ* = 0.3738 Å, beam size ~2.0 × 2.0 μm^2^) of the EBS-ESRF. In order to determine the sample position for single-crystal X-ray diffraction data acquisition, a full X-ray diffraction mapping of the pressure chamber was performed. The sample positions displaying the greatest number of single-crystal reflections belonging to the phases of interest were chosen, and step-scans of 0.5° from −36° to +36° ω were performed. The CrysAlis^Pro^ software^40^ was utilized for the single-crystal data analysis. To calibrate the instrumental model in the CrysAlis^Pro^ software, *i.e*. the sample-to-detector distance, detector’s origin, offsets of the goniometer angles, and rotation of both the X-ray beam and detector around the instrument axis, we used a single crystal of orthoenstatite [(Mg_1.93_Fe_0.06_)(Si_1.93_,Al_0.06_)O_6_, *Pbca* space group, *a* = 8.8117(2) Å, *b* = 5.1832(10) Å, and *c* = 18.2391(3) Å]. The DAFi program^17^ was used for the search of reflections’ groups belonging to individual single-crystal domains. The crystal structures were then solved and refined using the OLEX2^41^ and JANA2006 software^42^. The crystallite sizes were estimated from X-ray maps. The crystallographic information is available in Supplementary Tables 2–6.

### Density functional theory calculations

First-principles calculations were performed using the framework of density functional theory (DFT) as implemented in the Vienna Ab initio Simulation Package (VASP)^43^. The Projector-Augmented-Wave (PAW) method^44,45^ was used to expand the electronic wave function in plane waves. The Generalized Gradient Approximation (GGA) functional was used for calculating the exchange-correlation energies, as proposed by Perdew–Burke–Ernzerhof (PBE)^46^. The PAW potentials with following valence configurations of 4*s*4*p*5*s*4*d* for Y, 4*f*6*s* for Dy, 3*p*4*s*3*d* for Fe, 3*s*3*p* for Cl, and 2*s*2*p* for C were used. The plane-wave kinetic energy cutoff was set to 600 eV. The crystal structure, ELF, and charge density maps visualization were made with the VESTA software^47^. The finite displacement method, as implemented in PHONOPY^33^, was used to calculate phonon frequencies and phonon band structures.

## Supplementary information

Yin_PR File

Supplementary Information

Description of Additional Supplementary Files

Supplementary data 1

Supplementary data 2

Supplementary data 3

Supplementary data 4

## Data Availability

All data generated or analyzed during this study are included in this published article (and its Supplementary information files). The X-ray crystallographic coordinates for structures reported in this article have been deposited at the Cambridge Crystallographic Data Centre (CCDC), under deposition number CSD-2184739, CSD 2184740, CSD 2184741 and 2184742. These data can be obtained free of charge from The Cambridge Crystallographic Data Centre via www.ccdc.cam.ac.uk/data_request/cif.
