# Supplementary material for: Synthesis of rare-earth metal compounds through enhanced reactivity of alkali halides at high pressures
Source: Commun Chem. 2022 Oct 8;5:122. doi: 10.1038/s42004-022-00736-x (PMC9814685; doi:10.1038/s42004-022-00736-x)
Supplement: Supplementary file 2 — Supplementary Information [file 42004_2022_736_MOESM2_ESM.pdf]

Supplementary Information

Synthesis of Rare-Earth Metal Compounds through Enhanced Reactivity of Alkali Halides at High Pressures

Yuqing Yin[1,2*], Fariia I. Akbar[1,3], Elena Bykova[3,4], Alena Aslandukova[3], Dominique Laniel[5], Andrey Aslandukov[1,3], Maxim Bykov[6], Michael Hanfland[7], Gaston Garbarino[7], Zhitai Jia[2], Leonid Dubrovinsky[3], Natalia Dubrovinskaia[1,8]

Affiliations:

[1]Material Physics and Technology at Extreme Conditions, Laboratory of Crystallography, University of Bayreuth, 95440 Bayreuth, Germany

[2]State Key Laboratory of Crystal Materials, Shandong University, Jinan 250100, China

[3]Bayerisches Geoinstitut, University of Bayreuth, 95440 Bayreuth, Germany

[4]Earth and Planets Laboratory, Carnegie Institution for Science, 5241 Broad Branch Road, NW, Washington DC, 20015, USA

[5]Centre for Science at Extreme Conditions and School of Physics and Astronomy, University of Edinburgh, EH9 3FD Edinburgh, United Kingdom

[6]Institute of Inorganic Chemistry, University of Cologne, Greinstrasse 6, 50939 Cologne, Germany

[7]European Synchrotron Radiation Facility, B.P.220, F-38043 Grenoble Cedex, France

[8]Department of Physics, Chemistry and Biology (IFM), Linköping University, SE-581 83, Linköping, Sweden

*Corresponding author. Email: Yuqing.Yin@uni-bayreuth.de (Y.Y.)

Supplementary Tables

Supplementary Table 1. Summary of the experiments on high-pressure high-temperature synthesis in a diamond anvil cell.

DAC number	Starting materials	Pressure (GPa, ±1)	Measured temperature (K, ±200)	Reaction products
1	Y and NaCl	41	2000	Y_2ClC* + Y_2Cl + YC + Y_3C_4
2	Dy and NaCl	40	2000	Dy_2ClC* + DyCl + Dy_3C_4
3	Re and NaCl	38	2100	Reaction was observed, but the products were not identified
4	Ag and NaCl	44	1950	No reaction observed
5	FeO and KCl	160	2100	$FeCl_2$

*Carbon in the products of the reactions originates from diamond anvils.

Supplementary Table 2. Crystal structure, data collection and refinement details of Y_2Cl at 41(1) GPa in comparison to the corresponding DFT-relaxed structure.

**Crystal data**				**DFT results**
Chemical formula	Y_2Cl			Y_2Cl
M_r	213.27			
Crystal system, space group	Tetragonal, *I*4/*mcm*			Tetragonal, *I*4/*mcm*
Temperature (K)	293			
Pressure (GPa)	41(1)			40
a, *c* (Å)	6.1279 (3), 5.405 (7)			6.1480, 5.4205
V (Å^3)	202.96 (3)			204.88
Z	4			4
Radiation type	Synchrotron, λ = 0.41015 Å			
μ (mm^{-1})	13.77			
Crystal size (mm)	0.001 × 0.001 × 0.001			
**Data collection**				
Diffractometer	ESRF ID15b, EIGER2 X 9M CdTe detector			
Absorption correction (essentially for absorption in diamonds and pressure medium)	Multi-scan *CrysAlis PRO* 1.171.40.67a (Rigaku Oxford Diffraction, 2019) Empirical absorption correction using spherical harmonics, implemented in SCALE3 ABSPACK scaling algorithm.			
T_{min}, T_{max}	0.547, 1			
No. of measured, independent and observed [$I > 2\sigma(I)$] reflections	284, 104, 99			
R_{int}	0.026			
$(\sin \theta/\lambda)_{max}$ (Å^{-1})	0.887			
**Refinement**				
$R[F^2 > 2\sigma(F^2)]$, $wR(F^2)$, S	0.038, 0.100, 1.16			
No. of reflections	104			
No. of parameters	7			
$\Delta\rho_{max}$, $\Delta\rho_{min}$ (e Å^{-3})	2.42, -2.10			
**Crystal Structure**				
Atom	Wyckoff	Coordinates	U_{iso} (Å^2)	Coordinates (x y z)

<table>
<tr><td></td><td>Site</td><td>(x, y, z)</td><td></td><td></td></tr>
<tr><td>Y1</td><td>8h</td><td>0.6586(7) 0.1586(7) 1/2</td><td>0.0090(4)</td><td>0.65855 0.15855 1/2</td></tr>
<tr><td>Cl1</td><td>4a</td><td>0 0 1/4</td><td>0.0095(5)</td><td>0 0 1/4</td></tr>
<tr><td colspan="4">Anisotropic displacement parameters</td><td></td></tr>
</table>

<table>
<tr><td>Atom</td><td>U_{11} (Å^2)</td><td>U_{22} (Å^2)</td><td>U_{33} (Å^2)</td><td>U_{12} (Å^2)</td><td>U_{13} (Å^2)</td><td>U_{23} (Å^2)</td><td></td></tr>
<tr><td>Y1</td><td>0.0056(4)</td><td>0.0056(4)</td><td>0.0157(7)</td><td>0.00025(10)</td><td>0</td><td>0</td><td></td></tr>
<tr><td>Cl1</td><td>0.0063(6)</td><td>0.0063(6)</td><td>0.0158(14)</td><td>0</td><td>0</td><td>0</td><td></td></tr>
</table>

Supplementary Table 3. Crystal structure, data collection and refinement details of DyCl at 40(1) GPa in comparison to the corresponding DFT-relaxed structure.

**Crystal data**				**DFT results**
Chemical formula	DyCl			DyCl
M_r	197.95			
Crystal system, space group	Hexagonal, $P6_3/mmc$			Hexagonal, $P6_3/mmc$
Temperature (K)	293			
Pressure (GPa)	40(1)			40
a, c (Å)	3.0787 (19), 7.621 (5)			3.0922, 7.6267
V (Å^3)	62.56 (9)			63.15
Z	2			2
Radiation type	Synchrotron, λ = 0.3738 Å			
μ (mm^{-1})	11.32			
Crystal size (mm)	0.001 × 0.001 × 0.001			
**Data collection**				
Diffractometer	ESRF ID27, EIGER2 X CdTe 9M detector			
Absorption correction (essentially for absorption in diamonds and pressure medium)	Multi-scan *CrysAlis PRO* 1.171.41.120a (Rigaku Oxford Diffraction, 2021) Empirical absorption correction using spherical harmonics, implemented in SCALE3 ABSPACK scaling algorithm.			
T_{min}, T_{max}	0.617, 1			
No. of measured, independent and observed [$I > 2\sigma(I)$] reflections	90, 44, 41			
R_{int}	0.018			
$(\sin \theta/\lambda)_{max}$ (Å^{-1})	0.689			
**Refinement**				
$R[F^2 > 2\sigma(F^2)]$, $wR(F^2)$, S	0.045, 0.104, 1.24			
No. of reflections	44			
No. of parameters	5			
$\Delta\rho_{max}$, $\Delta\rho_{min}$ (e Å^{-3})	2.23, -5.92			
**Crystal Structure**				
	Wyckoff	Coordinates	U_{iso} (Å^2)	Coordinates (x y z)

		Site	(x, y, z)				
Dy1		2*a*	0 0 1/2		0.0091(8)		0 0 1/2
Cl1		2*c*	1/3 2/3 3/4		0.0115(10)		1/3 2/3 3/4
**Anisotropic displacement parameters**							
Atom	U_{11} (Å^2)	U_{22} (Å^2)	U_{33} (Å^2)	U_{12} (Å^2)	U_{13} (Å^2)	U_{23} (Å^2)	
Dy1	0.0103(9)	0.0103(9)	0.0066(12)	0.0052(5)	0	0	
Cl1	0.0127(13)	0.0127(13)	0.0090(17)	0.0064(7)	0	0	

Supplementary Table 4. Crystal structure details of $FeCl_2$ at 160(1) GPa in comparison to the corresponding DFT-relaxed structure.

**Crystal data**				**DFT results**
Chemical formula	$FeCl_2$			$FeCl_2$
M_r	126.75			
Crystal system, space group	Cubic, $Pa\bar{3}$			Cubic, $Pa\bar{3}$
Temperature (K)	293			
Pressure (GPa)	160(1)			150
a (Å)	4.8289 (11)			4.8280
V (Å^3)	112.60 (8)			112.54
Z	4			4
No. of measured, independent and observed [$I > 2\sigma(I)$] reflections	288, 91, 84			
R_{int}	0.023			
$(\sin \theta/\lambda)_{max}$ (Å^{-1})	0.879			
$R[F^2 > 2\sigma(F^2)]$, $wR(F^2)$, S	0.038, 0.103, 1.23			
No. of reflections	91			
No. of parameters	6			
$\Delta\rho_{max}$, $\Delta\rho_{min}$ (e Å^{-3})	1.15, -0.94			
**Crystal Structure**				
	Wyckoff Site	Coordinates (x, y, z)	U_{iso} (Å^2)	Coordinates (x y z)
Fe1	4*b*	0 1/2 0	0.0080(4)	0 1/2 0
Cl1	8*c*	0.14803(15) 0.14803(15) 0.14803(15)	0.0080(4)	0.14731 0.14731 0.14731

Supplementary Table 5. Crystal structure, data collection and refinement details of Y_2ClC at 41(1) GPa in comparison to the corresponding DFT-relaxed structure.

**Crystal data**				**DFT results**
Chemical formula	Y_2ClC			Y_2ClC
M_r	225.28			
Crystal system, space group	Trigonal, $R\bar{3}m$			Trigonal, $R\bar{3}m$
Temperature (K)	293			
Pressure (GPa)	41(1)			40
a, c (Å)	3.3690 (7), 17.703 (6)			3.3842, 17.8335
V (Å^3)	174.01 (10)			176.88
Z	3			3
Radiation type	Synchrotron, λ = 0.41015 Å			
μ (mm^{-1})	12.06			
Crystal size (mm)	0.001 × 0.001 × 0.001			
**Data collection**				
Diffractometer	ESRF ID15b, EIGER2 X 9M CdTe detector			
Absorption correction (essentially for absorption in diamonds and pressure medium)	Multi-scan *CrysAlis PRO* 1.171.40.67a (Rigaku Oxford Diffraction, 2019) Empirical absorption correction using spherical harmonics, implemented in SCALE3 ABSPACK scaling algorithm.			
T_{min}, T_{max}	0.299, 1			
No. of measured, independent and observed [$I > 2\sigma(I)$] reflections	117, 53, 51			
R_{int}	0.042			
$(\sin \theta/\lambda)_{max}$ (Å^{-1})	0.713			
**Refinement**				
$R[F^2 > 2\sigma(F^2)]$, $wR(F^2)$, S	0.050, 0.125, 1.20			
No. of reflections	53			
No. of parameters	8			
$\Delta\rho_{max}$, $\Delta\rho_{min}$ (e Å^{-3})	1.93, -1.74			
**Crystal Structure**				
	Wyckoff	Coordinates	U_{iso} (Å^2)	Coordinates (x y z)

<table>
<tr><td colspan="2"></td><td>Site</td><td colspan="2">(x, y, z)</td><td colspan="2"></td><td></td></tr>
<tr><td colspan="2">Y1</td><td>6c</td><td colspan="2">0 0 0 0.23654(9)</td><td colspan="2">0.0140(9)</td><td>0 0 0.23619</td></tr>
<tr><td colspan="2">Cl1</td><td>3a</td><td colspan="2">0 0 0</td><td colspan="2">0.0148(16)</td><td>0 0 0</td></tr>
<tr><td colspan="2">C1</td><td>3b</td><td colspan="2">0 0 1/2</td><td colspan="2">0.006(5)</td><td>0 0 1/2</td></tr>
<tr><td colspan="7">Anisotropic displacement parameters</td><td></td></tr>
<tr><td>Atom</td><td>U_{11} (Å^2)</td><td>U_{22} (Å^2)</td><td>U_{33} (Å^2)</td><td>U_{12} (Å^2)</td><td>U_{13} (Å^2)</td><td>U_{23} (Å^2)</td><td></td></tr>
<tr><td>Y1</td><td>0.0140(11)</td><td>0.0140(11)</td><td>0.0142(13)</td><td>0.0070(5)</td><td>0</td><td>0</td><td></td></tr>
<tr><td>Cl1</td><td>0.014(2)</td><td>0.014(2)</td><td>0.016(4)</td><td>0.0071(10)</td><td>0</td><td>0</td><td></td></tr>
<tr><td>C1</td><td>0.007(7)</td><td>0.007(7)</td><td>0.005(11)</td><td>0.003(3)</td><td>0</td><td>0</td><td></td></tr>
</table>

Supplementary Table 6. Crystal structure, data collection and refinement details of Dy_2ClC at 40(1) GPa in comparison to the corresponding DFT-relaxed structure.

**Crystal data**				**DFT results**
Chemical formula	Dy_2ClC			Dy_2ClC
M_r	372.46			
Crystal system, space group	Trigonal, $R\bar{3}m$			Trigonal, $R\bar{3}m$
Temperature (K)	293			
Pressure (GPa)	40(1)			40
a, c (Å)	3.3193 (7), 18.004 (10)			3.3743, 18.0156
V (Å^3)	171.79 (12)			177.64
Z	3			3
Radiation type	Synchrotron, λ = 0.3738 Å			
μ (mm^{-1})	11.80			
Crystal size (mm)	0.003 × 0.003 × 0.003			
**Data collection**				
Diffractometer	ESRF ID27, EIGER2 X CdTe 9M detector			
Absorption correction (essentially for absorption in diamonds and pressure medium)	Multi-scan *CrysAlis PRO* 1.171.41.120a (Rigaku Oxford Diffraction, 2021) Empirical absorption correction using spherical harmonics, implemented in SCALE3 ABSPACK scaling algorithm.			
T_{min}, T_{max}	0.64, 1			
No. of measured, independent and observed [$I > 2\sigma(I)$] reflections	121, 121, 120			
R_{int}	0.015			
$(\sin \theta/\lambda)_{max}$ (Å^{-1})	0.893			
**Refinement**				
$R[F^2 > 2\sigma(F^2)]$, $wR(F^2)$, S	0.037, 0.095, 1.16			
No. of reflections	121			
No. of parameters	8			
$\Delta\rho_{max}$, $\Delta\rho_{min}$ (e Å^{-3})	2.87, -2.34			
**Crystal Structure**				
	Wyckoff	Coordinates	U_{iso} (Å^2)	Coordinates (x y z)

<table>
<tr><td colspan="2"></td><td>Site</td><td colspan="2">(x, y, z)</td><td colspan="2"></td><td></td></tr>
<tr><td colspan="2">Dy1</td><td>6c</td><td colspan="2">0 0 0.23608(7)</td><td colspan="2">0.0108(5)</td><td>0 0 0.23595</td></tr>
<tr><td colspan="2">Cl1</td><td>3a</td><td colspan="2">0 0 0</td><td colspan="2">0.0115(15)</td><td>0 0 0</td></tr>
<tr><td colspan="2">C1</td><td>3b</td><td colspan="2">0 0 1/2</td><td colspan="2">0.010(5)</td><td>0 0 1/2</td></tr>
<tr><td colspan="7">Anisotropic displacement parameters</td><td></td></tr>
<tr><td>Atom</td><td>U_{11} (Å^2)</td><td>U_{22} (Å^2)</td><td>U_{33} (Å^2)</td><td>U_{12} (Å^2)</td><td>U_{13} (Å^2)</td><td>U_{23} (Å^2)</td><td></td></tr>
<tr><td>Dy1</td><td>0.0125(5)</td><td>0.0125(5)</td><td>0.0074(12)</td><td>0.0063(2)</td><td>0</td><td>0</td><td></td></tr>
<tr><td>Cl1</td><td>0.0152(18)</td><td>0.0152(18)</td><td>0.004(6)</td><td>0.0076(9)</td><td>0</td><td>0</td><td></td></tr>
</table>

Supplementary Table 7. Calculated enthalpies (eV) per Y atom of the $R\bar{3}m$ and $P6_3/mmc$[1] Y_2ClC.

Pressure	ΔH/Y atom ($R\bar{3}m$ Y_2ClC) (eV/atom)	ΔH/Y atom ($P6_3/mmc$ Y_2ClC) (eV/atom)
0	-14.2021	-14.2110
2	-13.7204	-13.7279
4	-13.2503	-13.2561
6	-12.7906	-12.7944
8	-12.3403	-12.3418
10	-11.8982	-11.8973
20	-9.7887	-9.7751
30	-7.8089	-7.7829
40	-5.9272	-5.8899

Supplementary Table 8. Unit cell parameters of the phases observed after laser-heating of Re and NaCl at 38(1) GPa (DAC 3, see Table S1).

Domain number	*a b c* (Å)	*α β γ* (°)	*V* (Å^3)
1	2.689(12) 9.225(14) 3.531(6)	90 90 90	87.58(4)
2	8.091(12) 7.138(11) 12.925(15)	90 95.05(3) 90	743.60(9)
3	4.803(7) 4.803(7) 4.803(7)	90 90 90	110.81(3)

Supplementary Table 9. Comparison of unit cell parameters (in hexagonal setting) and some bond lengths of $R\bar{3}m$ Y_2ClC, $R\bar{3}m$ Y_2Cl, $R\bar{3}m$ Dy_2ClC, and $R\bar{3}m$ Dy_2Cl at 40 GPa.

DFT model	Y_2ClC	Y_2Cl	DFT model	Dy_2ClC	Dy_2Cl
Space group	$R\bar{3}m$	$R\bar{3}m$	Space group	$R\bar{3}m$	$R\bar{3}m$
a (Å)	3.38	3.10	*a* (Å)	3.37	3.08
c (Å)	17.83	19.17	*c* (Å)	18.02	19.37
V (Å^3)	176.88	159.91	V (Å^3)	177.64	159.42
Y1-Y1 (Å)	3.157	3.057	Dy1-Dy1 (Å)	3.166	3.059
Y1-Y2 (Å)	3.384	3.104	Dy1-Dy2 (Å)	3.374	3.083
Y1-Y3 (Å)	3.978	4.304	Dy1-Dy3 (Å)	4.013	4.35
Y1-Cl1 (Å)	2.611	2.653	Dy1-Cl1 (Å)	2.622	2.666
Y1-C1 (Å)	2.314	-	Dy1-C1 (Å)	2.314	-
Y1-e^- (Å)*	-	2.178	Dy1-e^- (Å)*	-	2.171

*indicates that the position of e^- is the center of anionic electrons.

Supplementary Figures

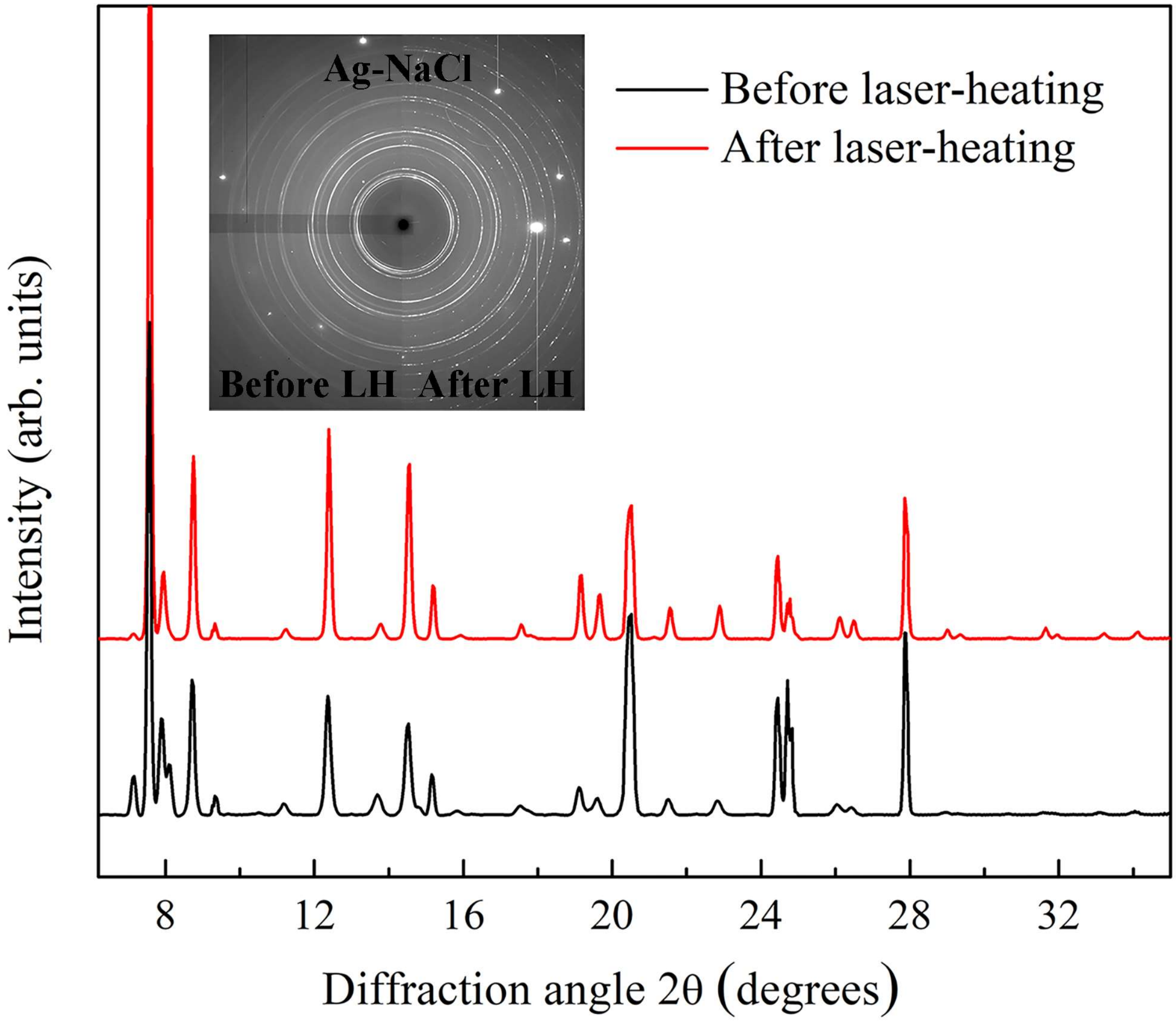

Supplementary Figure 1. Powder X-ray diffraction patterns of the Ag-NaCl sample before (black) and after (red) laser-heating at ~1950 K and 44 GPa. The patterns are similar, giving no indication of a chemical reaction. 2D XRD patterns in the inset show that the sample re-crystallized after laser heating, the single-crystal data we obtained from the laser-heated area are only of the cubic Ag and B2-NaCl.

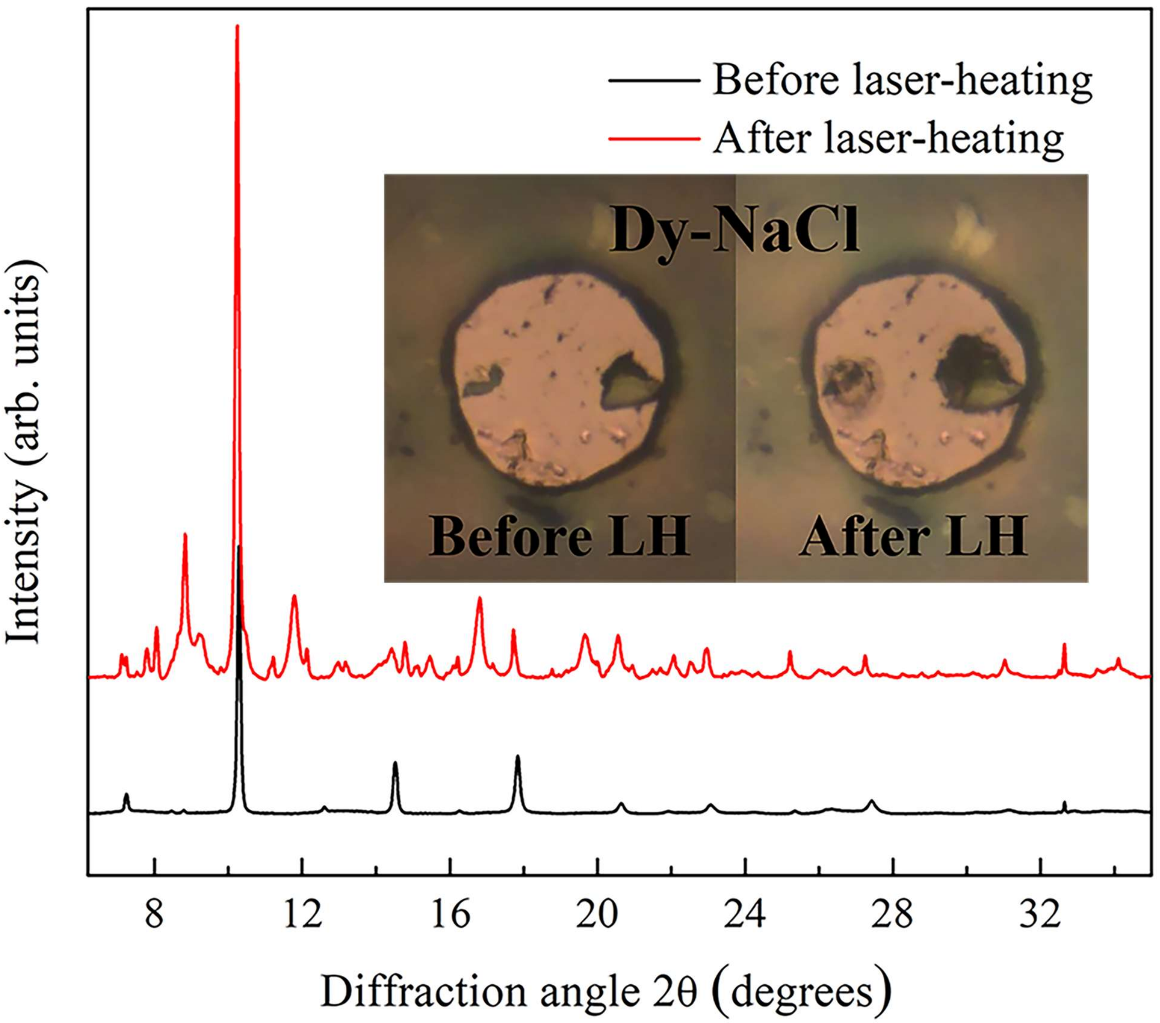

Supplementary Figure 2. Powder X-ray diffraction patterns of the Dy-NaCl sample before (black) and after (red) laser-heating at ~2000 K and 40 GPa. Inserts are optical photographs of the Dy-NaCl sample, demonstrating the production of additional products around the laser-heated area.

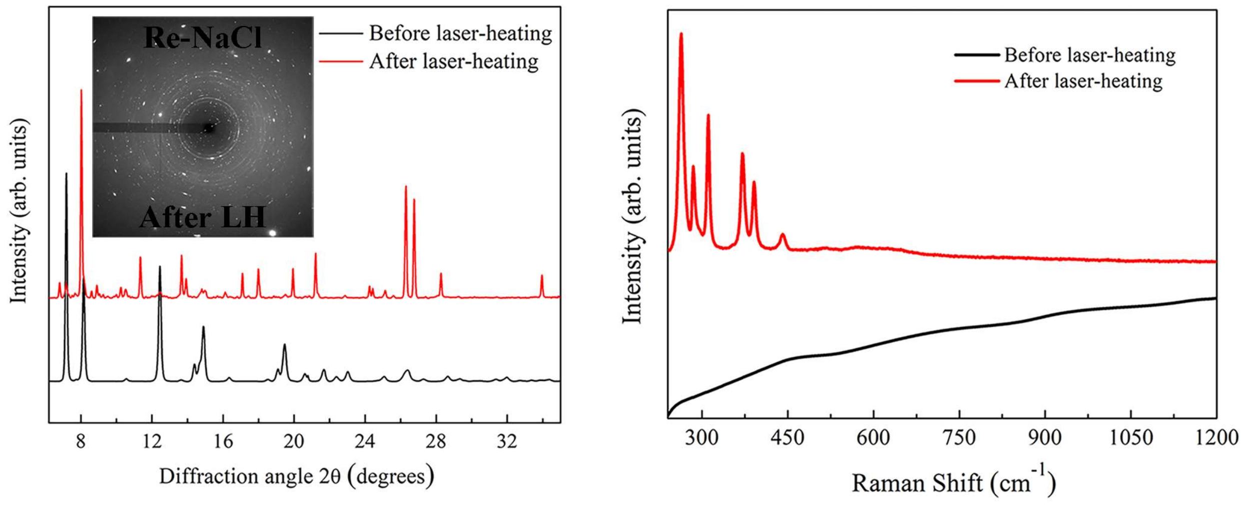

(a) (b)

Supplementary Figure 3. (a) Powder X-ray diffraction patterns of the Re-NaCl sample before (black) and after (red) laser-heating at ~2100 K and 38 GPa. Insert shows a 2D XRD pattern of the sample after its laser heating. (b) Raman spectrum of the Re-NaCl sample before and after laser-heating. Both the XRD pattern and the Raman spectrum give evidence of the chemical alteration of the sample. Using single-crystal X-ray diffraction data, we were able to determine the unit cell parameters of the new phases (see Supplementary Table 8). However, no reliable structural solutions were obtained.

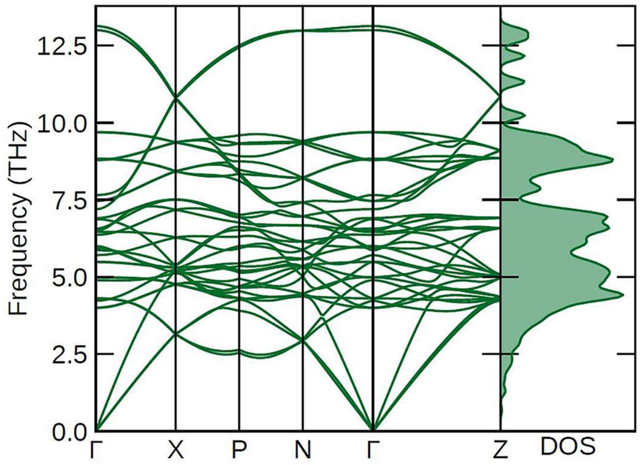

Supplementary Figure 4. Phonon dispersion curves along high-symmetry directions in the Brillouin zone and phonon density of states for Y_2Cl calculated at 40 GPa.

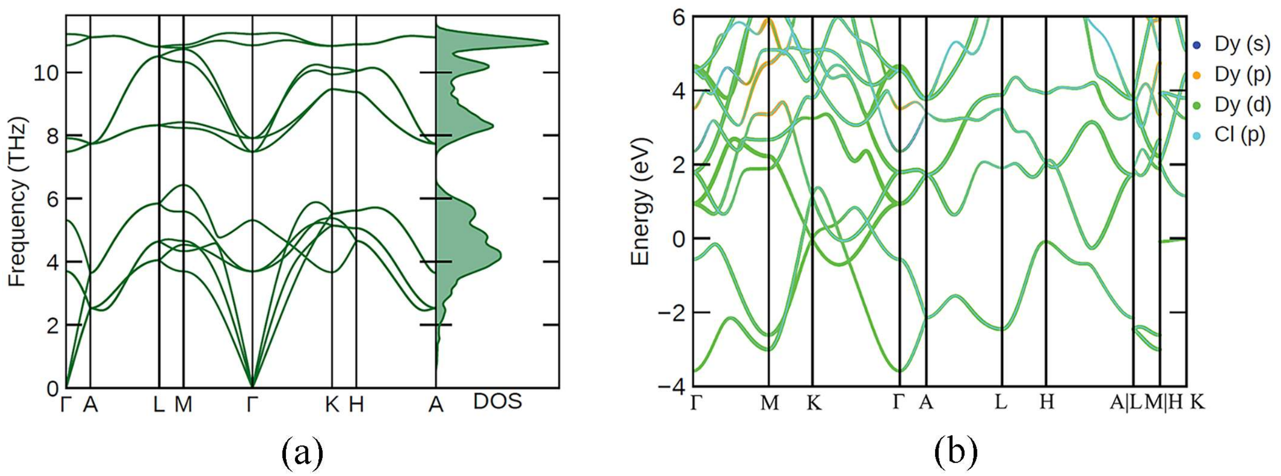

Supplementary Figure 5. (a) Phonon dispersion curves along high-symmetry directions in the Brillouin zone and phonon density of states for DyCl calculated at 40 GPa; (b) Calculated band structure of DyCl at 40 GPa. The Fermi energy level was set to 0 eV.

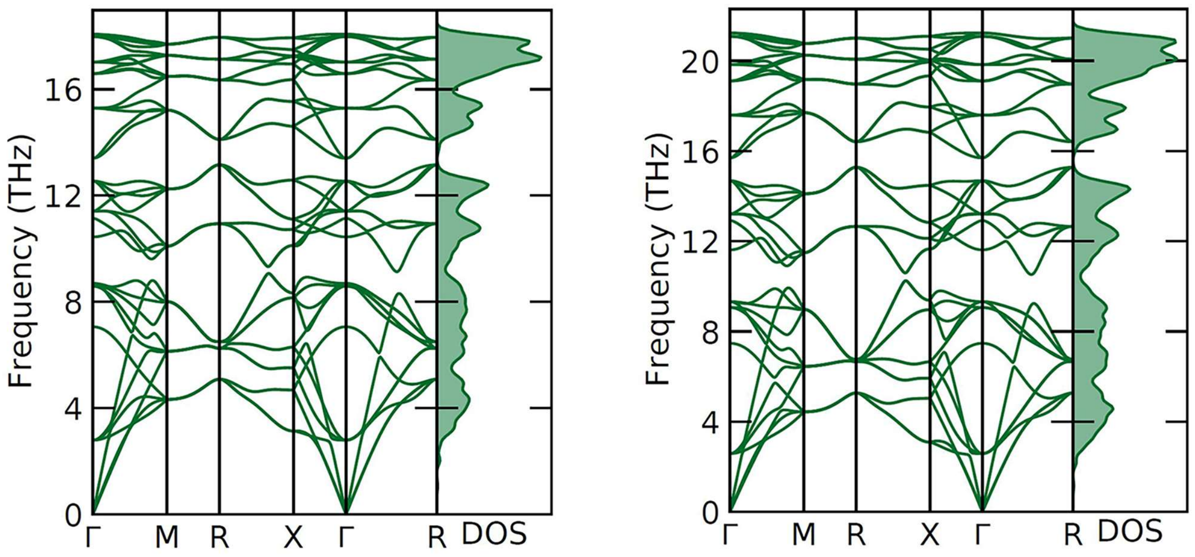

(a)

(b)

Supplementary Figure 6. Phonon dispersion curves along high-symmetry directions in the Brillouin zone and phonon density of states for $FeCl_2$ calculated at (a) 90 GPa and (b) 150 GPa.

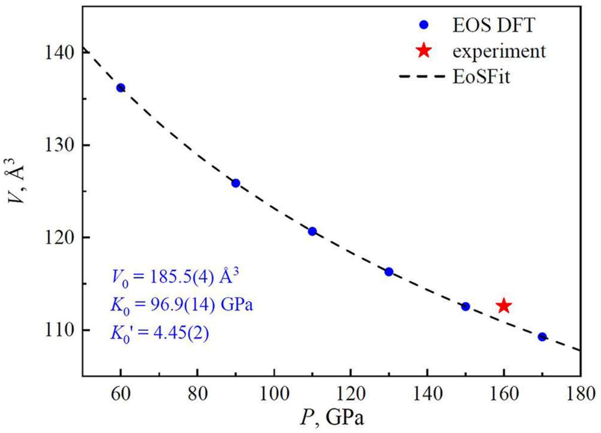

Supplementary Figure 7. Calculated (blue) and experimental (red) pressure-volume dependence of $Pa\bar{3}$ $FeCl_2$. Dashed lines represent the third-order Birch Murnaghan fit of the results.

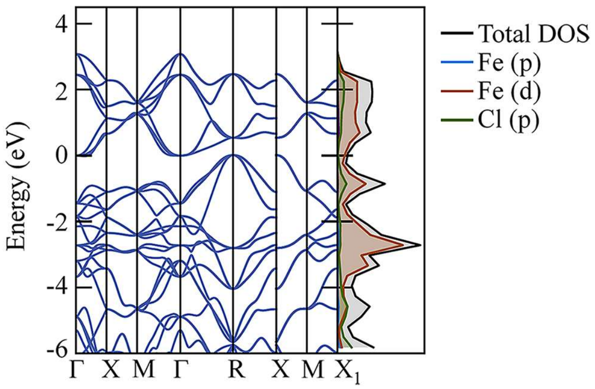

(a)

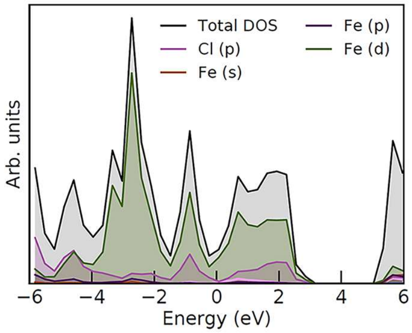

(b)

Supplementary Figure 8. (a) Calculated band structure and electron density of states of $FeCl_2$ at 150 GPa; (b) TDOS and PDOS curves of $FeCl_2$ at 150 GPa. The Fermi energy level was set to 0 eV.

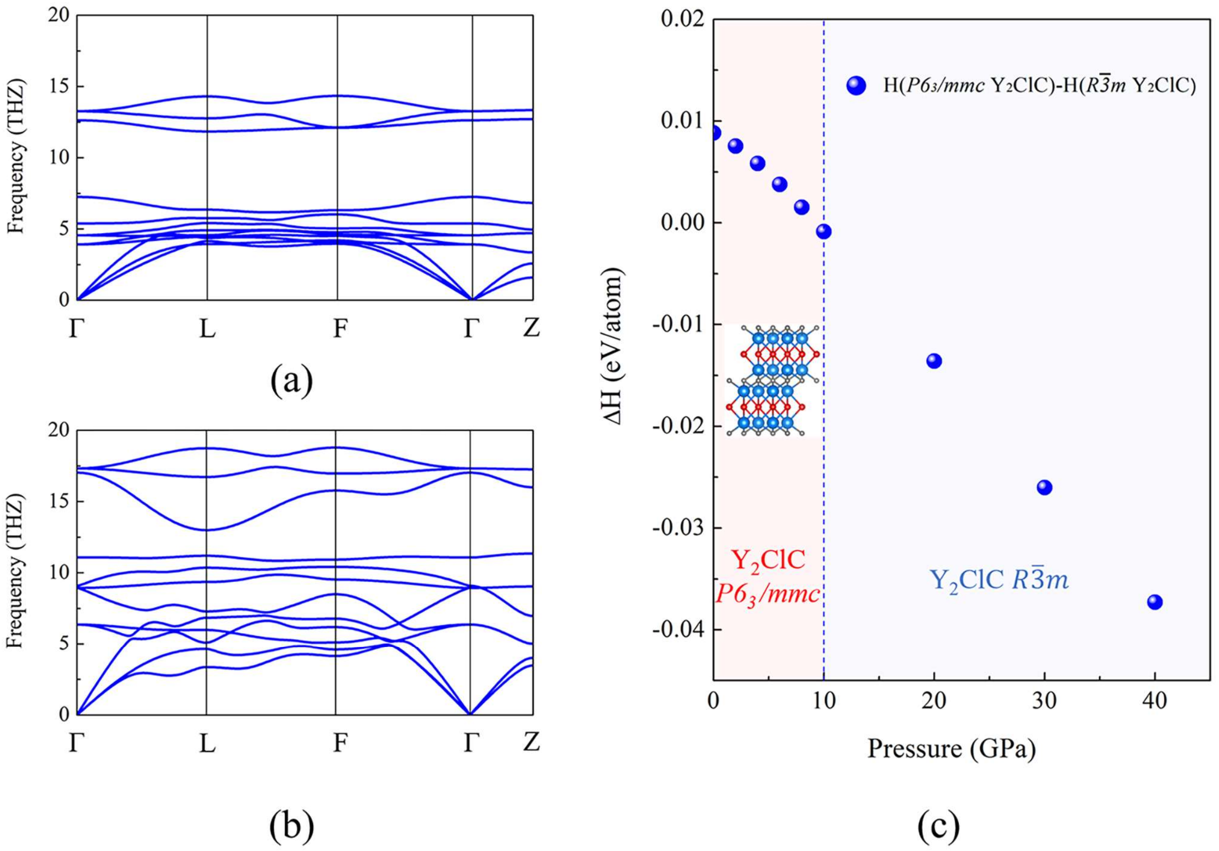

Supplementary Figure 9. Phonon dispersion curves along high-symmetry directions in the Brillouin zone for $R\bar{3}m$ Y_2ClC calculated at (a) 0 GPa and (b) 40 GPa. (c) The calculated difference in enthalpy per Y atom ($\Delta H = H(P6_3/mmc\ Y_2ClC) - H(R\bar{3}m\ Y_2ClC)$) as a function of pressure.

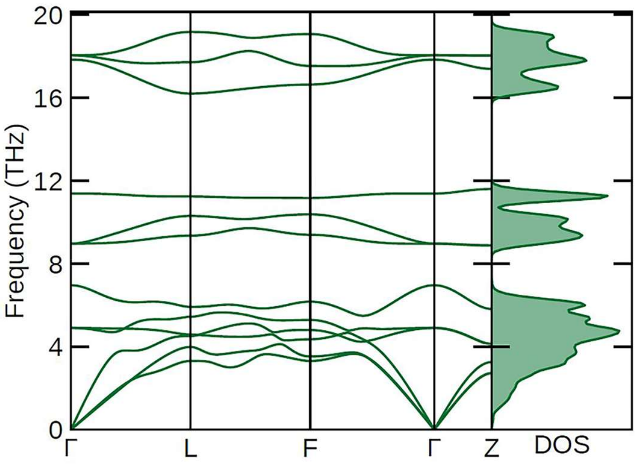

Supplementary Figure 10. Phonon dispersion curves along high-symmetry directions in the Brillouin zone and phonon density of states for $R\overline{3}m$ Dy_2ClC calculated at 40 GPa.

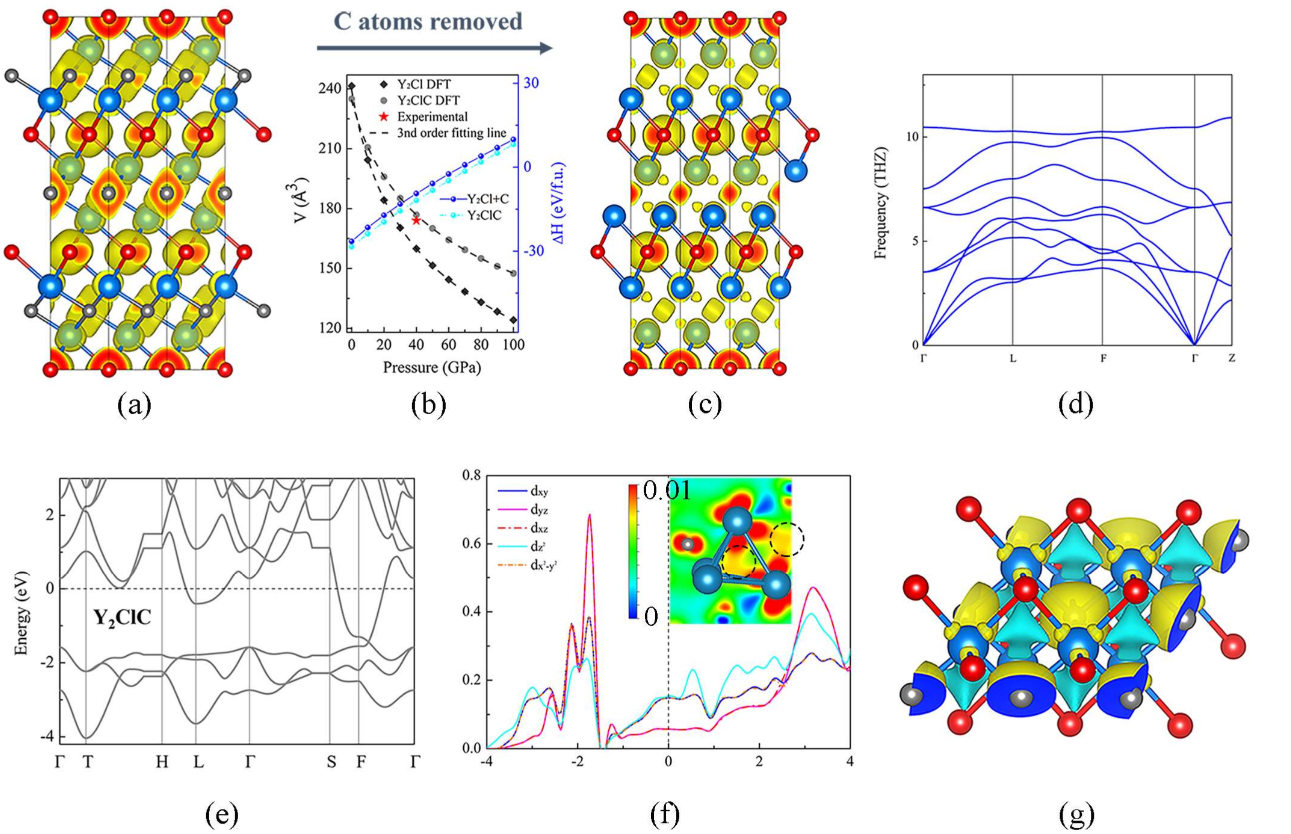

Supplementary Figure 11. (a) The electron localization function (ELF) of Y_2ClC with the isosurfaces value of 0.6; (b) The pressure dependence of the unit cell volume of $R\bar{3}m$ Y_2Cl and Y_2ClC (with left axis) and the calculated enthalpies per formula unit (f.u.) of the reactants and products of reaction $Y_2Cl + C \rightarrow Y_2ClC$ (with right axis); (c) ELF of $R\bar{3}m$ Y_2Cl with the isosurfaces value of 0.6; (d) Phonon dispersion curves along high-symmetry directions in the Brillouin zone and phonon density of states for $R\bar{3}m$ Y_2Cl calculated at 40 GPa; (e) Calculated band structure of $R\bar{3}m$ Y_2ClC at 40 GPa. The Fermi energy level was set to 0 eV. (f) The PDOS curves of the Y-*d* orbitals. The Fermi energy level was set to 0 eV. Insert shows theY_4 tetrahedron and the partial charge density map in the (1 0 -1) plane (rhombohedral setting) around the Fermi level (-1 eV < $E-E_F$ < 0 eV). The black circles emphasize the Y-*d* orbital overlapping in the Y_4 tetrahedron. (g) Charge density difference for C atoms inserted in $R\bar{3}m$ Y_2Cl. Yellow indicates regions of charge gain and blue indicates regions of charge loss.

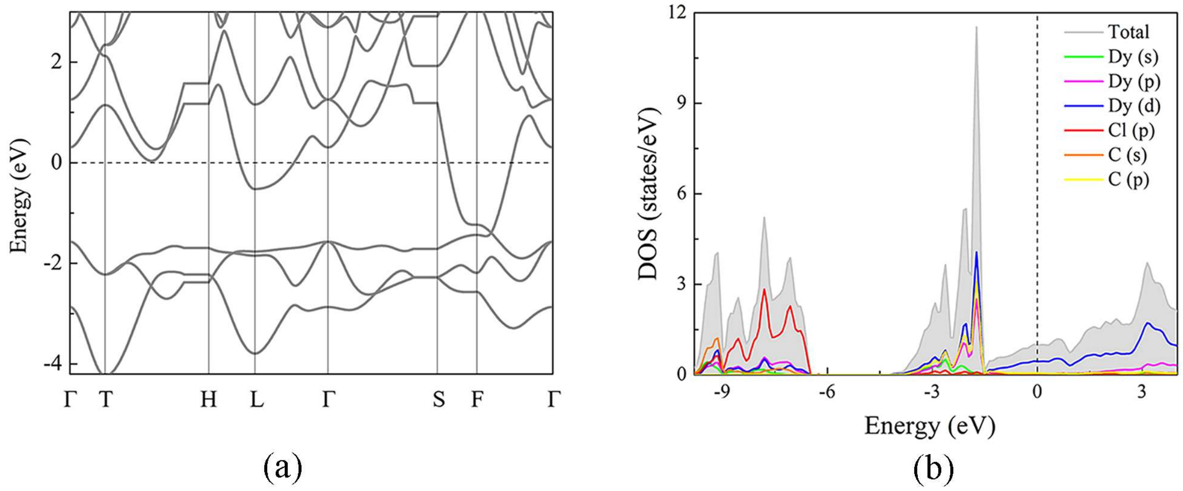

Supplementary Figure 12. (a) Calculated band structure of Dy_2ClC at 40 GPa; (b) TDOS and PDOS curves of Dy_2ClC at 40 GPa. The Fermi energy level was set to 0 eV.

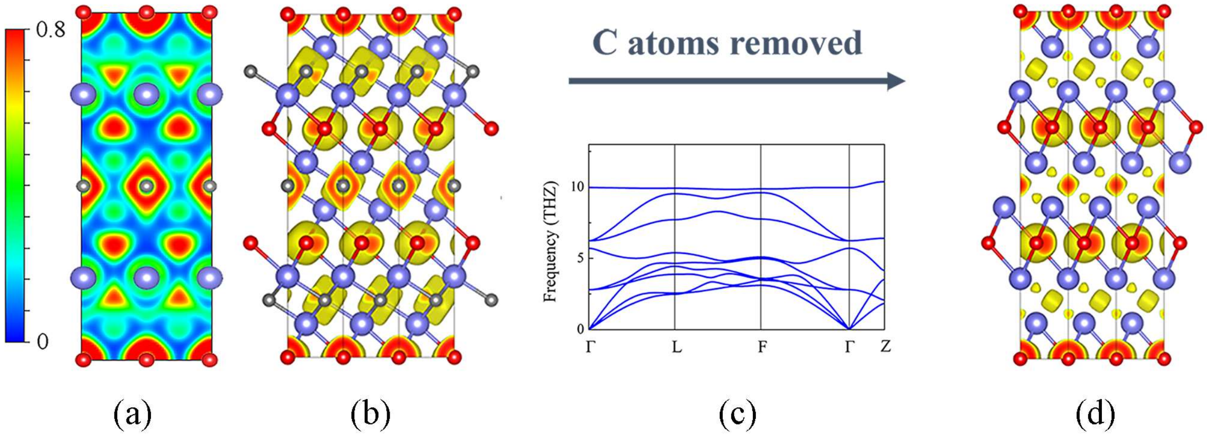

Supplementary Figure 13. (a) The 2D ELF map of Dy_2ClC (1 0 0) plane; (b) ELF of Dy_2ClC with the isosurfaces value of 0.6; (c) Phonon dispersion curves along high-symmetry directions in the Brillouin zone for $R\overline{3}m$ Dy_2Cl calculated at 40 GPa; (d) ELF of $R\overline{3}m$ Dy_2Cl with the isosurfaces value of 0.6.

Supplementary Discussion

Electronic properties of Y_2ClC and Dy_2ClC.

With the C atoms removed in this novel $R\bar{3}m$ Y_2ClC compound, DFT calculations succeeded in optimizing this novel $R\bar{3}m$ Y_2Cl structure. The anionic electrons (Supplementary Figure 11c) perfectly localized in the place of the C atoms in Y_2ClC (Supplementary Figure 11a). The equation of states (EoS) of Y_2ClC and Y_2Cl together with the enthalpies of reactants and product of the reaction ($Y_2Cl + C \rightarrow Y_2ClC$) were shown in Supplementary Figure 11b, which further explained that in the experiments we synthesized the Y_2ClC compound. Notably, the electride $R\bar{3}m$ Y_2Cl is also a dynamically stable structure at 40 GPa (Supplementary Figure 11d). Like the $R\bar{3}m$ YCl studied by Wan et al.[2], $R\bar{3}m$ Y_2Cl also has two ELF attractors in the centers of the Y_4 tetrahedra and Y_6 octahedra (Supplementary Figure 11c). It is obvious that even though the introduction of the C layers in $R\bar{3}m$ Y_2ClC elongates the Y-Y bond length, the electron localization in the Y_4 tetrahedra caused by the *d*-orbital overlapping still exist (Supplementary Figure 11 e-f). An intuitive display of the charge transfer after the introduction of C atoms can be obtained from Supplementary Figure 11g, which is consistent with the above analysis. Introduction of the C atoms causes charge loss in the blue region (Y_4 tetrahedra) and charge gain in the yellow region (Y_6 octahedra), leaving a weak electron bridge in Figure 4b.

The band structure and the DOS curves of Dy_2ClC (Supplementary Figure 12) show a very close distribution pattern with Y_2ClC. The hybridization of the Dy-*d* orbitals disperses electrons at the Fermi level (E_F), leading to the metallic phase. Similar features of the electron bridges in ELF induced by the Dy *d*-orbital are shown in Supplementary Figure 13. Likewise, if the C atoms are removed, the dynamically stable structure $R\bar{3}m$ Dy_2Cl (Supplementary Figure 13c) will be obtained after the structure optimization at 40 GPa.

The introduction of C atoms clearly affects the bond length in the layered structure (see Supplementary Table 9). Due to the larger volume of C atoms, the distances between the first (Y1/Dy1-Y1/Dy1) and second (Y1/Dy1-Y2/Dy2) neighboring Y/Dy atoms become longer with the addition of C atoms, thus providing a larger Y_6/Dy_6 octahedra volume for C atoms. But the metal-Cl bond lengths (Y1/Dy1-Cl1) and the distances between the two metal layers (Y1/Dy1-Y3/Dy3) connecting the chlorine atoms become smaller. This also leads to the compression of the unit cell parameters on the c-axis after the introduction of C atoms, which means that there is a stronger binding force between the layers, making Y_2ClC/Dy_2ClC a more favoured phase.

Supplementary References

1. Ohmer, D., Qiang, G., Opahle, I., Singh, H. K. & Zhang, H. High-throughput design of 211−M2AX compounds. *Phys. Rev. Mater.* **3**, 053803, doi:10.1103/PhysRevMaterials.3.053803 (2019).
2. Wan, B. *et al.* Identifying quasi-2D and 1D electrides in yttrium and scandium chlorides via geometrical identification. *Npj Comput Mater* **4**, 77, doi:10.1038/s41524-018-0136-1 (2018).
