# Supplementary material for: Synthesis of rare-earth metal compounds through enhanced reactivity of alkali halides at high pressures
Source: Commun Chem. 2022 Oct 8;5:122. doi: 10.1038/s42004-022-00736-x (PMC9814685; doi:10.1038/s42004-022-00736-x)
Supplement: Supplementary file 3 — Description of Additional Supplementary Files [file 42004_2022_736_MOESM3_ESM.docx]

Description of Additional Supplementary Files

**File name:** Supplementary Data 1

**Description:** Cif file of Y2Cl.

**File name:** Supplementary Data 2

**Description:** Cif file of DyCl.

**File name:** Supplementary Data 3

**Description:** Cif file of Y2ClC.

**File name:** Supplementary Data 4

**Description:** Cif file of Dy2ClC.
