# Supplementary material for: Synthesis of rare-earth metal compounds through enhanced reactivity of alkali halides at high pressures
Source: Commun Chem. 2022 Oct 8;5:122. doi: 10.1038/s42004-022-00736-x (PMC9814685; doi:10.1038/s42004-022-00736-x)
Supplement: Supplementary file 1 — Yin_PR File [file 42004_2022_736_MOESM1_ESM.pdf]

nature portfolio

Peer Review File

Synthesis of Rare-Earth Metal Compounds through Enhanced Reactivity of Alkali Halides at High Pressures

Open Access This file is licensed under a Creative Commons Attribution 4.0 International License, which permits use, sharing, adaptation, distribution and reproduction in any medium or format, as long as you give appropriate credit to the original author(s) and the source, provide a link to the Creative Commons license, and indicate if changes were made. In the cases where the authors are anonymous, such as is the case for the reports of anonymous peer reviewers, author attribution should be to 'Anonymous Referee' followed by a clear attribution to the source work. The images or other third party material in this file are included in the article's Creative Commons license, unless indicated otherwise in a credit line to the material. If material is not included in the article's Creative Commons license and your intended use is not permitted by statutory regulation or exceeds the permitted use, you will need to obtain permission directly from the copyright holder. To view a copy of this license, visit http://creativecommons.org/licenses/by/4.0/.

Reviewers' comments:

Reviewer #1 (Remarks to the Author):

Manuscript “Synthesis of Novel Rare-Earth Metal Compounds through Enhanced Reactivity of Alkali Halides at High Pressures” reports new chlorides and chloride carbides synthesized at about 40GPa and 2000K. Structures of these compounds were solved and refined with in-situ single-crystal X-ray diffraction technique using synchrotron radiation. This manuscript requires some minor revision before publishing.

1. Detailed descriptions of new crystal structures should be mostly moved to supplementary materials while still focusing on the most essential structural information in the main text, including whether a structural type is known and any unusual crystal chemical features.

2. Methods. X-ray diffraction. It would be useful if authors include beam size at the sample position and whether data from multiple crystals have been combined.

3. Tables S2-S6.
3.1. Resolution limits (smallest d-values) must be included.
3.2. How crystal sizes were estimated?
3.3. Absorption correction. Data sets from such small microcrystals themselves in general don’t require absorption correction. Authors should clarify that they implemented correction essentially for absorption in diamonds and pressure medium.
3.4. Authors must clarify whether refinements were performed in anisotropic or isotropic approach of thermal parameters.
3.5. Line 286: is reference for DAFi program correct?
3.6. Table S4: no information on structure refinement is given at all! If authors detected this structure without doing refinement it must be clarified. Uncertainties presented for positions of Cl are very confusing in this case.

Reviewer #2 (Remarks to the Author):

This paper reports unusual phenomena in DAC experiments namely the reaction of metals (and FeO) with NaCl and KCl media. This is quite unexpected and raises questions about both experiments and computations. The results and the interpretations are reliable although the complex mixture of the reactants and products certainly causes challenges. The topic and the message carried by the paper fit Communication Chemistry very well. Just a couple of minor points for the discussion.

1. The observed metal chlorides and carbochlorides can only be the results of the reaction with NaCl and KCl media. The paper didn’t discuss where the alkali and alkaline metals go after the reaction. Is it possible to figure out the reaction equation?
2. The ELF maxima can be seen in Y2ClC. IS this compound a high-pressure electride?

Reviewer #3 (Remarks to the Author):

The authors present evidence that probably support the formation of Y2Cl and DyCl and Y2ClC and Dy2ClC at ~ 40 GPa and 2000 K in a laser heated DAC. However, the novelty of this results is limited. There are plenty of examples where similar chemical reactions were observed in the past. This has been known for more than 20 years. Indeed, in the same paper the authors commented on the formation of PdF2 [Earth. Am. Mineral. 107, 313-317 (2022). Not only it, articles about the reaction of metals with solid pressure-transmitting media have been published since long ago; Journal of Physics

and Chemistry of Solids Volume 70, 2009, 1117-1120. The reactions have been also described in a recent review of the laser heating method, Crystals 2020, 10, 459. On top of it, some of the authors of the present article discussed the subject in an article where they proposed to use of pulse laser heating instead of continuous laser heating; Minerals 2020, 10, 542. More examples can be found in Crystals 2019, 9, 676 (Reaction of sample contained with Re and W gaskets) and Geoscience Frontiers, 11, 1755-1761 (2020) (Reaction of Fe with solid pressure media). In summary, I considered the novelty and impact of this article limited. The results could be published in other journal, but not in Nature. The article does not meet the high-quality standards of Nature.

Response to referees' comments:

To better structure our response to the referees' reports, each of their comments has been copied and our response to each of these comments is written in blue.

Reviewer #1 (Remarks to the Author):

Manuscript "Synthesis of Novel Rare-Earth Metal Compounds through Enhanced Reactivity of Alkali Halides at High Pressures" reports new chlorides and chloride carbides synthesized at about 40GPa and 2000K. Structures of these compounds were solved and refined with in-situ single-crystal X-ray diffraction technique using synchrotron radiation. This manuscript requires some minor revision before publishing.

The authors thank the referee for his/her thorough review of the manuscript, insightful comments and suggestions.

1. Detailed descriptions of new crystal structures should be mostly moved to supplementary materials while still focusing on the most essential structural information in the main text, including whether a structural type is known and any unusual crystal chemical features.

The authors greatly respect referee's suggestion. We indeed put all detailed descriptions, including tables with full crystallographic data and cif files, in supplementary materials. The crystal structure descriptions in the text are not lengthy, in our opinion, and include only that information, which is important for discussing the crystal chemistry of the novel compounds. Concerning the most essential structural information, such as structural types and unusual crystal chemical features, it is provided in the main text in detail.

2. Methods. X-ray diffraction. It would be useful if authors include beam size at the sample position and whether data from multiple crystals have been combined.

As suggested by the referee, the beam size information has been included in Methods.
The structure of each compound was solved and refined using the data from one single crystal which had the best data quality. No combination of data from multiple crystals has been performed.

The sentence in Methods was modified as follows:
"Synchrotron X-ray diffraction measurements of the compressed samples were performed at ID15 (λ = 0.41015 Å, beam size ~ 5.0 × 5.0 μm^2) and ID27 (λ = 0.3738 Å, beam size ~ 2.0 × 2.0 μm^2) of the EBS-ESRF."

3. Tables S2-S6.
3.1. Resolution limits (smallest d-values) must be included.

We would like to say that the resolution limits are presented in Table S2-S6 in the form of $(\sin\theta/\lambda)_{max}$. The smallest d-values can be calculated from $1/[2*(\sin\theta/\lambda)_{max}]$.

3.2. How crystal sizes were estimated?

The crystallite sizes were estimated from X-ray maps. We have added this information in Methods.

3.3. Absorption correction. Data sets from such small microcrystals themselves in general don't require absorption correction. Authors should clarify that they implemented correction essentially for absorption in diamonds and pressure medium.

We thank the referee. We have added this information.

3.4. Authors must clarify whether refinements were performed in anisotropic or isotropic approach of thermal parameters.

As suggested by the referee, we have added the anisotropic displacement parameters to Table S2 (Y_2Cl), Table S3 (DyCl), Table S5 (Y_2ClC), and Table S6 (Dy_2ClC).

3.5. Line 286: is reference for DAFi program correct?

We thank the referee for pointing this out. We have corrected the reference number for DAFi program (ref. 17 in the revised manuscript). (Aslandukov, A., Aslandukov, M., Dubrovinskaia, N. & Dubrovinsky, L. Domain Auto Finder (DAFi) program: the analysis of single-crystal X-ray diffraction data from polycrystalline samples. *Journal of Applied Crystallography*, in press (2022).)

3.6. Table S4: no information on structure refinement is given at all! If authors detected this structure without doing refinement it must be clarified. Uncertainties presented for positions of Cl are very confusing in this case.

We apologize for incomplete Table S4. It has been completed.

Reviewer #2 (Remarks to the Author):

This paper reports unusual phenomena in DAC experiments namely the reaction of metals (and FeO) with NaCl and KCl media. This is quite unexpected and raises questions about both experiments and computations. The results and the interpretations are reliable although the complex mixture of the reactants and products certainly causes challenges. The topic and the message carried by the paper fit Communication Chemistry very well. Just a couple of minor points for the discussion.

We would like to thank the referee for his/her positive comments.

1. The observed metal chlorides and carbochlorides can only be the results of the reaction with NaCl and KCl media. The paper didn't discuss where the alkali and alkaline metals go after the reaction. Is it possible to figure out the reaction equation?
Currently we do not know the reactions pathways. More experimental data is needed to figure out the reaction equations in the situation, when Na- or K-containing products were not detected. For

this reason, any discussion might have been only speculative, but not affecting our results or conclusions, so that we just abstained of any reasonings.

2. The ELF maxima can be seen in Y_2ClC. IS this compound a high-pressure electride?

This is indeed an interesting question. We cannot exclude that this material may be an electride like YCl [npj Computational Materials (2018) 4:77], but we cannot answer more certain on this question based only on the ELF maxima that can be seen in Y_2ClC. This requires additional considerations which are beyond our expertise and out of scope of the current paper.

Reviewer #3 (Remarks to the Author):

The authors present evidence that probably support the formation of Y_2Cl and DyCl and Y_2ClC and Dy_2ClC at ~ 40 GPa and 2000 K in a laser heated DAC. However, the novelty of this results is limited. There are plenty of examples where similar chemical reactions were observed in the past. This has been known for more than 20 years. Indeed, in the same paper the authors commented on the formation of PdF_2 [Earth. Am. Mineral. 107, 313-317 (2022). Not only it, articles about the reaction of metals with solid pressure-transmitting media have been published since long ago; Journal of Physics and Chemistry of Solids Volume 70, 2009, 1117-1120. The reactions have been also described in a recent review of the laser heating method, Crystals 2020, 10, 459. On top of it, some of the authors of the present article discussed the subject in an article where they proposed to use of pulse laser heating instead of continuous laser heating; Minerals 2020, 10, 542. More examples can be found in Crystals 2019, 9, 676 (Reaction of sample contained with Re and W gaskets) and Geoscience Frontiers, 11, 1755-1761 (2020) (Reaction of Fe with solid pressure media). In summary, I considered the novelty and impact of this article limited. The results could be published in other journal, but not in Nature. The article does not meet the high-quality standards of Nature.

We strongly object this referee. Neither of his/her claims is true and neither of the references given by the referee #3 is relevant to our work. The detailed arguments have been provided by referee #2 in the letter to editor.
